# Serum proteomic profiling reveals MTA2 and AGO2 as potential prognostic biomarkers associated with disease activity and adverse outcomes in multiple myeloma

**DOI:** 10.1371/journal.pone.0278464

**Published:** 2022-12-01

**Authors:** Dollapak Apipongrat, Sittiruk Roytrakul, Kannadit Prayongratana, Mongkon Charoenpitakchai, Kamphon Intharanut, Chonlada Laoruangroj, Panachai Silpsamrit, Oytip Nathalang

**Affiliations:** 1 Graduate Program in Biomedical Sciences, Faculty of Allied Health Sciences, Thammasat University, Bangkok, Pathumthani, Thailand; 2 Division of Hematology, Department of Medicine, Phramongkutklao Hospital, Bangkok, Thailand; 3 Functional Ingredients and Food Innovation Research Group, National Center for Genetic Engineering and Biotechnology, National Science and Technology Development Agency, Khlong Luang, Pathumthani, Thailand; 4 Department of Pathology, Pramongkutklao College of Medicine, Bangkok, Thailand; Universitat des Saarlandes, GERMANY

## Abstract

Multiple myeloma (MM) is an incurable plasma cell malignancy accounting for approximately 10% of hematological malignancies. Identification of reliable biomarkers for better diagnosis and prognosis remains a major challenge. This study aimed to identify potential serum prognostic biomarkers corresponding to MM disease activity and evaluate their impact on patient outcomes. Serum proteomic profiles of patients with MM and age-matched controls were performed using LC–MS/MS. In the verification and validation phases, the concentration of the candidate biomarkers was measured using an ELISA technique. In addition, the association of the proposed biomarkers with clinical outcomes was assessed. We identified 23 upregulated and 15 downregulated proteins differentially expressed in newly diagnosed and relapsed/refractory MM patients compared with MM patients who achieved at least a very good partial response to treatment (≥VGPR). The top two candidate proteins, metastasis-associated protein-2 (MTA2) and argonaute-2 (AGO2), were selected for further verification and validation studies. Both MTA2 and AGO2 showed significantly higher levels in the disease-active states than in the remission states (*p* < 0.001). Regardless of the patient treatment profile, high MTA2 levels were associated with shorter progression-free survival (*p* = 0.044; HR = 2.48; 95% CI, 1.02 to 6.02). Conversely, high AGO2 levels were associated with IgG and kappa light-chains isotypes and an occurrence of bone involvement features (*p* < 0.05) and were associated with prolonged time to response (*p* = 0.045; HR = 3.00; 95% CI, 1.03 to 8.76). Moreover, the analytic results using a publicly available NCBI GEO dataset revealed that AGO2 overexpression was associated with shorter overall survival among patients with MM (*p* = 0.032, HR = 1.60, 95% CI, 1.04 to 2.46). In conclusion, MTA2 and AGO2 proteins were first identified as potential biomarkers that reflect disease activity, provide prognostic values and could serve as non-invasive indicators for disease monitoring and outcome predicting among patients with MM.

## Introduction

Multiple myeloma (MM) is a plasma cell neoplasm characterized by the clonal proliferation of malignant plasma cells within the bone marrow (BM), which is accompanied by monoclonal protein (M-protein) in blood or urine and associated organ dysfunctions [1]. MM is the second most common hematological malignancy, accounting for approximately 10% of all cases [1, 2]. The incidence rates of MM range from 1.5 to 6.0 per 100,000 person-years and are strongly related to age and sex [3, 4]. The median age at diagnosis is 69 years, and the median survival rate is 5 to 7 years for patients receiving a new diagnosis of MM [5, 6]. Currently, advanced therapeutic options in MM treatment have markedly improved, resulting in increasingly lengthened patient’s survival. However, MM is still considered an incurable disease, mainly due to the inevitable emergence of relapsed/refractory disease, and its mechanism remains unknown [5, 7].

The diagnosis and risk stratification of MM depends on traditional biomarkers, including M-protein, ß2-microglobulin, albumin, serum free-light chain (SFLC), lactate dehydrogenase (LDH), imaging techniques and cytogenetic abnormalities. In addition, the International Staging System (ISS) and the Revised International Staging System (R-ISS) have recently been used to stage patients [8]. Although several established biomarkers for MM have been identified, novel biomarkers that can reliably indicate disease activity, progression and patient outcomes are needed. Nevertheless, identifying these biomarkers remains a major challenge [9–11].

During the past few years, global expression analysis of proteins or proteomics has been widely used in many cancer research studies, including MM [12–19]. Proteomics provides the molecular machinery of cell physiology, including the level of protein expression, protein variations or isoforms, posttranslational modification and protein–protein interactions, providing superior advantages over genomic-based assays [19].

In MM, proteomic analysis has been used for several specific purposes, including studying advanced pathogenesis, identifying novel drug-targeting molecules, and identifying potential diagnostic and prognostic biomarkers [9–11]. Several studies have demonstrated the use of the proteomic approach to identify potential biomarkers in MM [14–18]. BM-derived proteomes, such as BM plasma cells and BM extracellular matrix proteomes, were commonly used as the particular protein source for biomarker discovery [14–16]. However, obtaining protein samples directly from the BM involves a highly invasive and painful procedure and is inappropriate for use as a routine biomarker in clinical practice. Hence, the use of liquid biopsies such as blood, serum or plasma, is a comprehensive approach due to its convenience, greater accessibility and containing all the biologically relevant information about the disease [11]. Serum or plasma comprises circulating tumor proteins and nucleic acids, including microRNAs (miRNAs) and cell-free DNA, which could reflect actual disease activity [11]. Moreover, these circulating biomarkers may provide a minimum residual disease indicating the occurrence of relapse in MM [11].

In this study, we performed serum proteomic profiles to identify potential prognostic biomarkers corresponding to MM disease activity and investigated the association between the proposed biomarkers and clinical outcomes among patients with MM.

## Materials and methods

### Patients and samples

**Fig 1:**
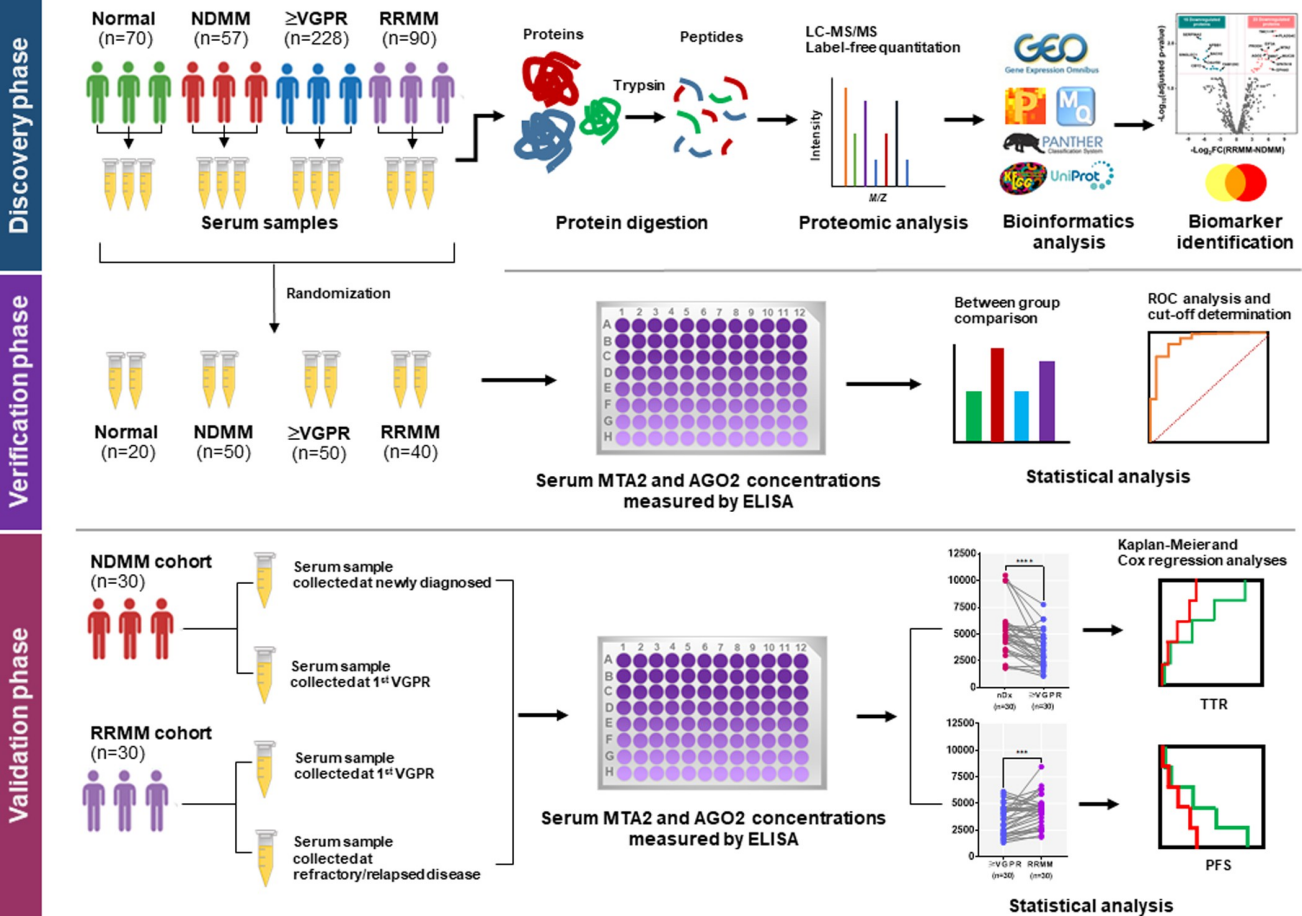
Schematic diagram demonstrating the experimental workflow in this study.

In the discovery phase, the serum samples of patients with MM, requested for routine serum protein electrophoresis (SPEP) analysis at the Special Hematology Laboratory, Department of Medicine, Phramongkutklao Hospital, Bangkok, Thailand, between May 2020 and December 2021 were included. In addition, 70 serum samples were obtained from age-matched unrelated healthy individuals (median age = 65.4 years) and used as normal controls. All serum samples were aliquoted and stored at −80°C until use. Overview of the experimental workflow employed in this study was divided in three phases, as shown in Fig 1. A total of 445 serum samples, including samples of patients with newly diagnosed MM (NDMM, n = 57), patients with MM who achieved at least a very good partial response to treatment (≥VGPR, n = 228), patients with relapsed/refractory MM (RRMM, n = 90) and normal controls (n = 70), were subjects for proteomic analysis. The demographic and clinical characteristics of the patients and controls are summarized in S1 Table in S1 File.

In the verification phase, the sample size was calculated using a pooled SD of log_2_ intensity from LC–MS/MS (SD of 17.6 and 18.4 for MTA2 and AGO2); a level of significance of 5% and a level of the estimation error of 5%. We estimated that at least 50 samples were required for each group. The simple randomized sampling was performed using a random sample of cases function in IBM SPSS Statistics for Windows, Version 20.0 (IBM Corp., Armonk, NY, USA), in which each sample has an exactly equal chance of being selected. However, due to insufficient volume existing in some samples, only 160 samples from NDMM (n = 50), ≥VGPR (n = 50), RRMM (n = 40) and normal controls (n = 20) were used for MTA2 and AGO2 measurement by enzyme-linked immunosorbent assay (ELISA).

In the validation phase, independent cohorts of 30 patients with NDMM and 30 with RRMM were enrolled. Paired serum samples were collected when newly diagnosed and ≥VGPR for the NDMM cohort, and when ≥VGPR and diagnosis of relapsed/refractory for the RRMM cohort. The demographic and clinical data of the patients included in the validation study are shown in S2 Table in S1 File. Clinical events consisting of disease progression, relapsed/refractory disease and death were recorded by prospective investigation. The patient’s response was assessed according to the International Myeloma Working Group (IMWG) response criteria [20, 21]. This study protocol was approved by the Institutional Review Board, Royal Thai Army Medical Department, Bangkok, Thailand (approved No. IRBRTA 433/2563) and the Human Research Ethics Committee of Thammasat University (HREC-TUSc, COE No. 015/2564). Written informed consent was obtained from all subjects following the Declaration of Helsinki.

### Liquid chromatography–tandem mass spectrometry (LC–MS/MS)

The LC–MS/MS analysis was performed using an Ultimate 3000 Nano/Capillary LC System (Thermo Scientific, UK) coupled to a Hybrid Quadrupole Q-Tof Impact II™ (Bruker Daltonics, Germany) equipped with a nano-captive spray ion source. The LC–MS/MS analysis of each sample was performed in triplicate as described in the Supplementary Methods in S1 File.

### Protein identification and label-free quantitation

MaxQuant 1.6.6.0 was used to quantify the proteins in each serum sample using the Andromeda search engine to correlate MS/MS spectra to the Uniprot *Homo sapiens* database [22]. Label-free quantitation with MaxQuant’s standard settings was performed as described in the Supplementary Methods in S1 File. The protein false discovery rate (FDR) was set at 0.01 and estimated using reversed search sequences. The maximal number of modifications per peptide was set to 5. Maximum peptide intensities were log_2_ transformed and missing values were imputed with a constant value (zero) using Perseus Software [23]. The visualization and statistical analyses were conducted using the MultiExperiment Viewer (MeV) in the TM4 Suite Software [24].

### Protein functional and pathway analyses

To explore the potential involving function and pathways of differentially expressed proteins, Protein Analysis Through Evolutionary Relationships (PANTHER, Version 11.1, available from: http://www.pantherdb.org/) was performed by keeping *Homo sapiens* as a selected organism [25]. Gene Ontology (GO) functional and Kyoto Encyclopedia of Genes and Genomes (KEGG) pathway enrichment analyses were performed. When the FDR was < 0.01, GO terms and KEGG pathways were significantly enriched. To analyze the common and forecasted functional interaction networks between identified proteins and small molecules, a protein–protein interaction (PPI) network was constructed using STITCH, Version 5.0 (available from: http://stitch.embl.de/) [26], and visualized by Cytoscape Software, V3.2 (1999 Free Software Foundation, Inc., Boston, MA, USA).

### Analysis of gene expression profiling and survival analysis

The potential involvement of the candidate proteins in MM was investigated in a parallel study using publicly available gene expression profiles (GEPs). The NCBI GEO microarray data set, accession no.: GSE47552 [27], was analyzed. Gene levels were expressed by normalized expression values and compared between the MM and control groups.

To evaluate the association of *MTA2* and *AGO2* gene expressions and patients with MM survival, a large GEP microarray dataset, GSE2658 [28], providing clinical outcomes for 350 patients with NDMM, was analyzed. Kaplan–Meier analysis was performed to compare the overall survival (OS) of patients with low *vs*. high expression levels of those proteins based on the median expression of the cohort.

### Enzyme-linked immunosorbent assay (ELISA)

The serum concentrations of MTA2 and AGO2 were measured using commercial ELISA kits; Human MTA2 [MBS2705865] and Human Proteins Argonaute-2/EIF2C2 [MBS910054] ELISA Kits (MyBioSource, CA, USA). The assay procedure was performed according to the manufacturer’s instructions. Each protein concentration was measured by comparing the optical density using a microplate reader (Synergy^TM^ HT, BioTek Instrument, VT, USA). The samples were measured in duplicate.

### Immunohistochemical (IHC) staining

The paraffin-embedded BM core biopsies obtained from patients with MM at NDMM (n = 3), at complete response (CR, n = 3), at diagnosis of RRMM (n = 3) and a non-cancerous BM (n = 1) were analyzed for the protein expression of MTA2 and AGO2. IHC staining was performed on a Ventana Discovery XT automated system (Ventana Medical System, Tucson, AZ, USA), using the primary antibody raised against MTA2 (diluted 1:500; ab8106, Abcam, UK) and AGO2 (diluted 1:200; ab226943, Abcam, UK), as described in the Supplementary Methods in S1 File. Two slides from each biopsy were stained with hematoxylin and eosin (H&E) for routine histological evaluation.

### Statistical analysis

Continuous variables were presented as mean, median, standard deviation (SD) and/or interquartile range (IQR) and compared using Student *t*-test, paired *t*-test, Mann–Whitney *U* test, Kruskal–Wallis test and ANOVA test. Categorical variables were described as frequency and percentage. The receiver operating characteristic (ROC) with the area under the curve (AUC) was used to determine the optimal cut-off providing high sensitivity and specificity for each protein. Clinical data and laboratory parameters were compared among diverse groups using Chi-Square (*χ*^*2*^) or Fisher’s exact tests, as appropriate. A Kaplan–Meier plot for time to response (TTR) and progression-free survival (PFS) was analyzed to determine the association between biomarkers and patients’ outcomes. The hazard ratio (HR) with a 95% confidence interval (CI) was calculated using univariate and/or multivariate Cox regression analysis. Statistical analysis was performed using IBM SPSS Statistics for Windows, Version 20.0 (IBM Corp., Armonk, NY, USA) and GraphPad Prism, Version 9 (GraphPad Software, CA, USA). A *p*-value of less than 0.05 was considered statistically significant.

## Results

### Discovery phase

#### Serum proteomic profiles of patients with MM and controls

**Fig 2:**
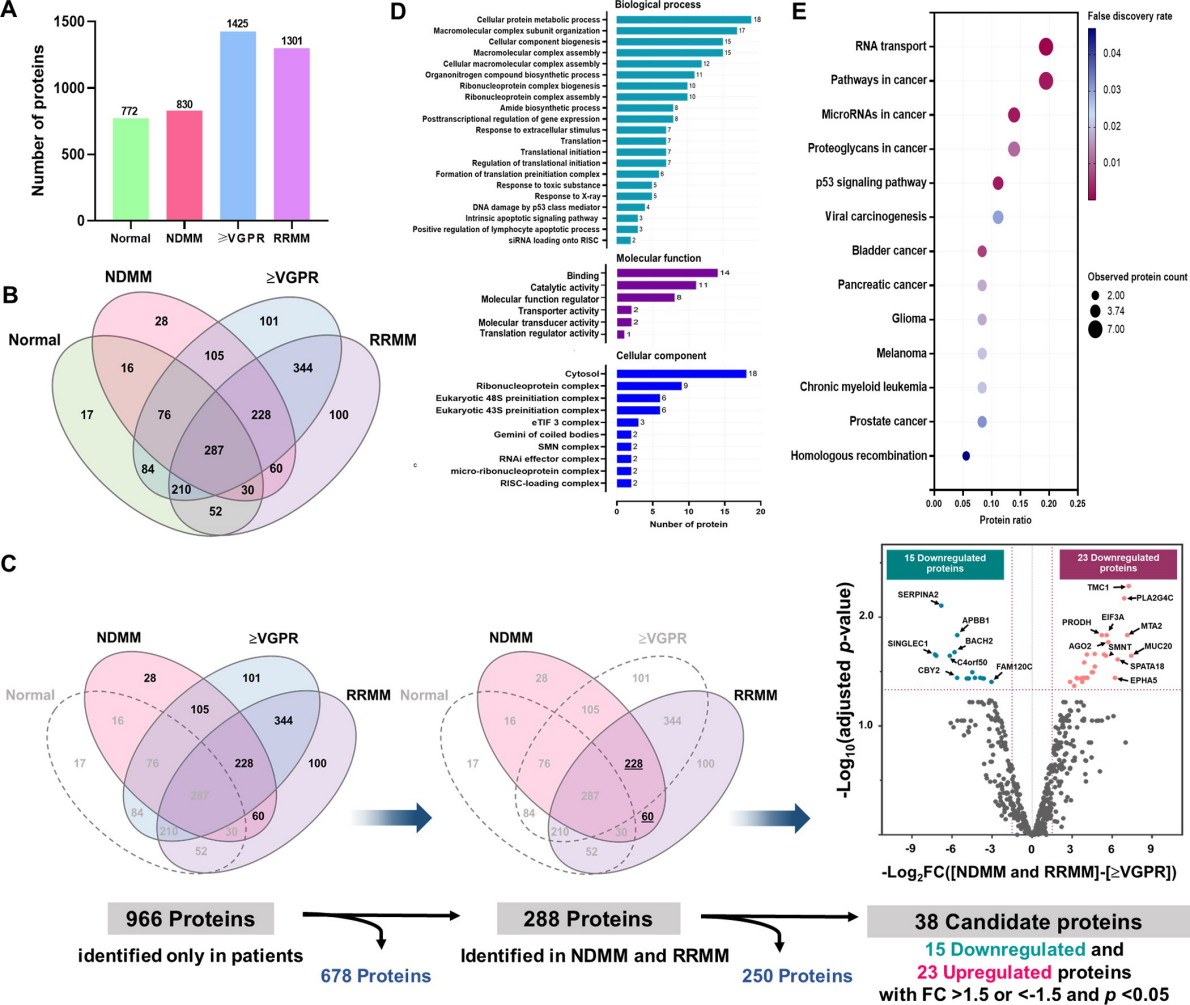
Differentially expressed proteins identified by LC-MS/MS in the discovery phase. (A) Bar chart demonstrating the number of serum proteins identified in each group. (B) Venn diagram illustrating the overlap between the serum proteins detected in normal, NDMM, ≥VGPR and RRMM. (C) The overview of candidate biomarker screening processes. Volcano plot demonstrating the change in the relative abundance of 288 proteins between disease-active states (NDMM and RRMM) and disease remission states (≥VGPR). The x-axis represents log_2_ fold changes of proteins, and the y-axis represents -log_10_ FDR-adjusted *p*-values. The rose pink and blue-green dots represent significantly upregulated and downregulated proteins with a log_2_-fold change >1.5 and <-1.5, respectively. (D) GO functional enrichment analysis of the 38 dysregulated proteins. Bar charts demonstrate the distribution according to their biological process, molecular function and cellular component. (E) KEGG pathway enrichment analysis of the 38 dysregulated proteins. The y-axis indicates the functional classification or pathway, and the x-axis indicates the protein ratio (observed protein count/total identified protein) of the respective pathway. The color key of the FDR and bubble size represent the observed protein count in the respective pathway.

Among the serum samples of 375 patients and 70 normal controls, 1,738 proteins were identified using LC–MS/MS with an FDR < 0.01. Of these, 830, 1,425, 1,301 and 772 proteins were identified in NDMM, ≥VGPR, RRMM and normal groups, respectively (Fig 2A). The overlap of the protein expressions among the study groups was illustrated using a Venn diagram (Fig 2B).

#### Identification of potential candidate biomarkers

Based on MM disease activity, the ideal biomarkers should be present in the active states of the disease, such as newly diagnosed or relapsed/refractory disease, but they exhibited downregulation or were absent in disease remission states (≥VGPR). Therefore, the differentially expressed proteins identified in normal groups (772 proteins) were filtered out. To identify potential biomarkers indicating MM disease activity, 966 differentially expressed proteins identified among patients were analyzed. Only 288 differentially expressed proteins identified in disease-active states (NDMM and RRMM) were compared with ≥VGPR (Fig 2C). Proteins with fold change (disease-active states *vs*. remission states) greater than 1.5 or less than -1.5 and adjusted *p*-values less than 0.05 were considered potential candidate biomarkers. Among these, 38 dysregulated proteins including 23 upregulated and 15 downregulated proteins were compatible with the criteria (Fig 2C). The upregulated and downregulated candidate proteins are listed in S3a and S3b Table in S1 File.

To evaluate the functional relevance of the 38 dysregulated proteins, GO functional and KEGG pathway enrichment analyses were performed. The proteins were classified based on their respective biological processes, molecular functions and cellular components (Fig 2D). The KEGG pathway enrichment analysis revealed that most dysregulated proteins were significantly enriched in RNA transport, pathways in cancer, miRNAs in cancer and p53 signaling pathway (Fig 2E).

**Fig 3:**
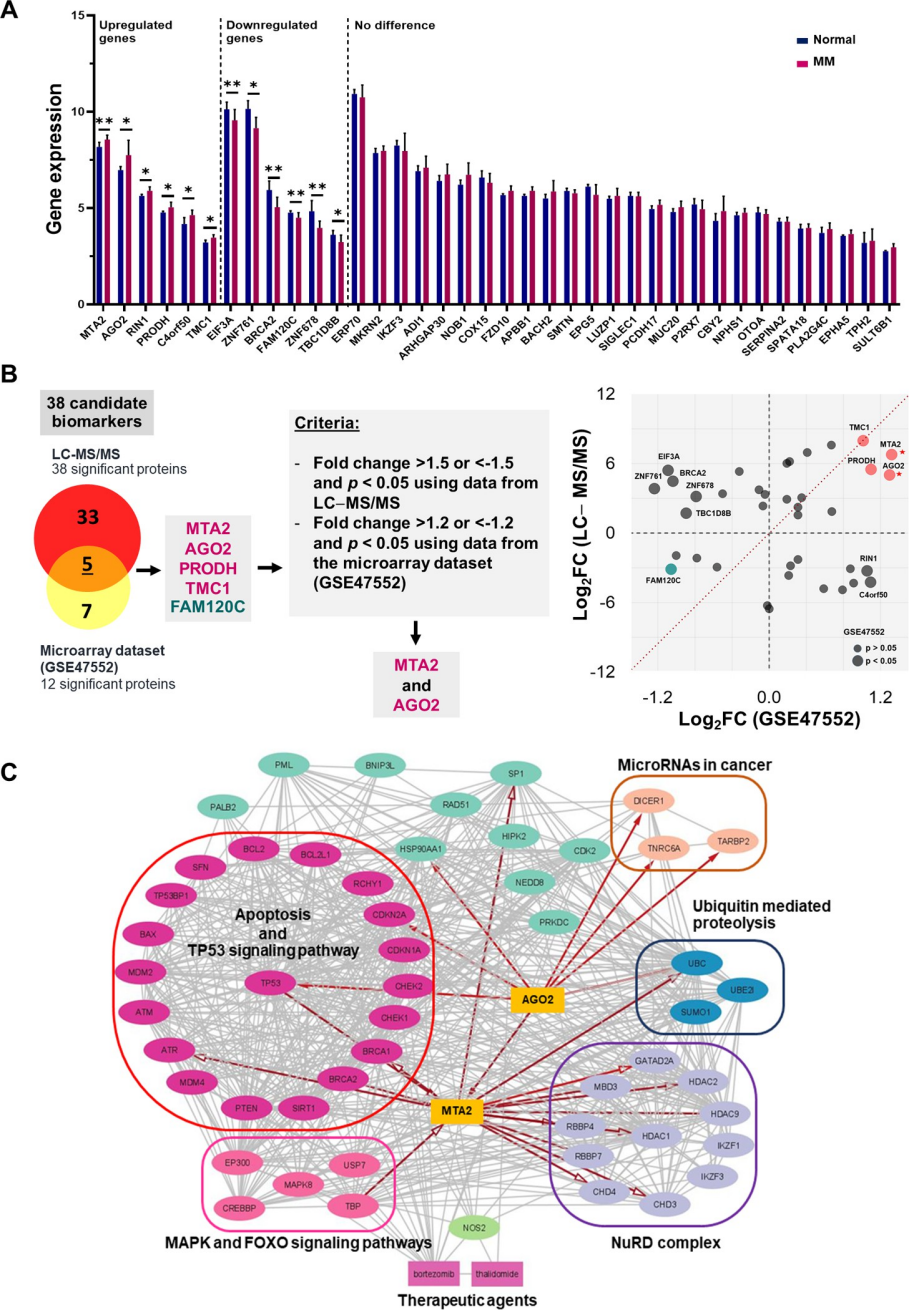
Analysis of gene expression profile corresponding to 38 dysregulated proteins and identifying of candidate biomarkers. (A) The expression profiles of the genes involving 38 dysregulated proteins among 41 patients with MM compared with 5 normal controls using the data from GSE47552 [27]. Bar chart showing the relative gene expression levels of genes involving 38 dysregulated proteins. The significant *p*-value less than 0.05 and 0.01 are represented with * and **, respectively. (B) Selecting of the candidate biomarkers. (C) KEGG pathway enrichment-based PPI network analysis. The top candidate proteins, MTA2 and AGO2 (gold-yellow), were predicted to be central mediators of multiple signaling pathways.

In addition, to confirm whether the dysregulated proteins were expressed by myeloma cells, the NCBI GEO microarray data set (accession no.GSE47552) of plasma cells isolated from 41 patients with MM and 5 normal controls [27] were analyzed. A comparison of normalized gene expression values among 38 dysregulated proteins encoding genes is shown in Fig 3A. The results revealed that 12 of 38 genes were significantly expressed among patients with MM. However, only five genes—metastasis-associated protein-2 (*MTA2*), argonaute-2 (*AGO2*), proline dehydrogenase (*PRODH*), transmembrane channel-like protein 1 (*TMC1*), and protein FAM120C (*FAM120C*)—showed significant expression patterns consistent with our results from LC–MS/MS. Regarding our criteria for biomarker selection consisting of (a) fold change >1.5 or <-1.5 and *p* < 0.05 by LC–MS/MS and (b) fold change >1.2 or <-1.2 and *p* < 0.05 using data from the microarray dataset [27], only MTA2 and AGO2 were selected for further analyses as potential biomarkers (Fig 3B).

#### KEGG enrichment-based network analysis of MTA2 and AGO2

Regarding KEGG enrichment-based PPI network analysis, MTA2 and AGO2 proteins were predicted as central mediators of multiple signaling pathways including apoptosis, miRNAs in cancer, ubiquitin-mediated proteolysis, nucleosome remodeling and deacetylase (NuRD) complex, p53, mitogen-activated protein kinases (MAPKs) and FOXO signaling pathways (Fig 3C).

### Verification phase

#### Verification of serum MTA2 and AGO2 levels by ELISA

**Fig 4:**
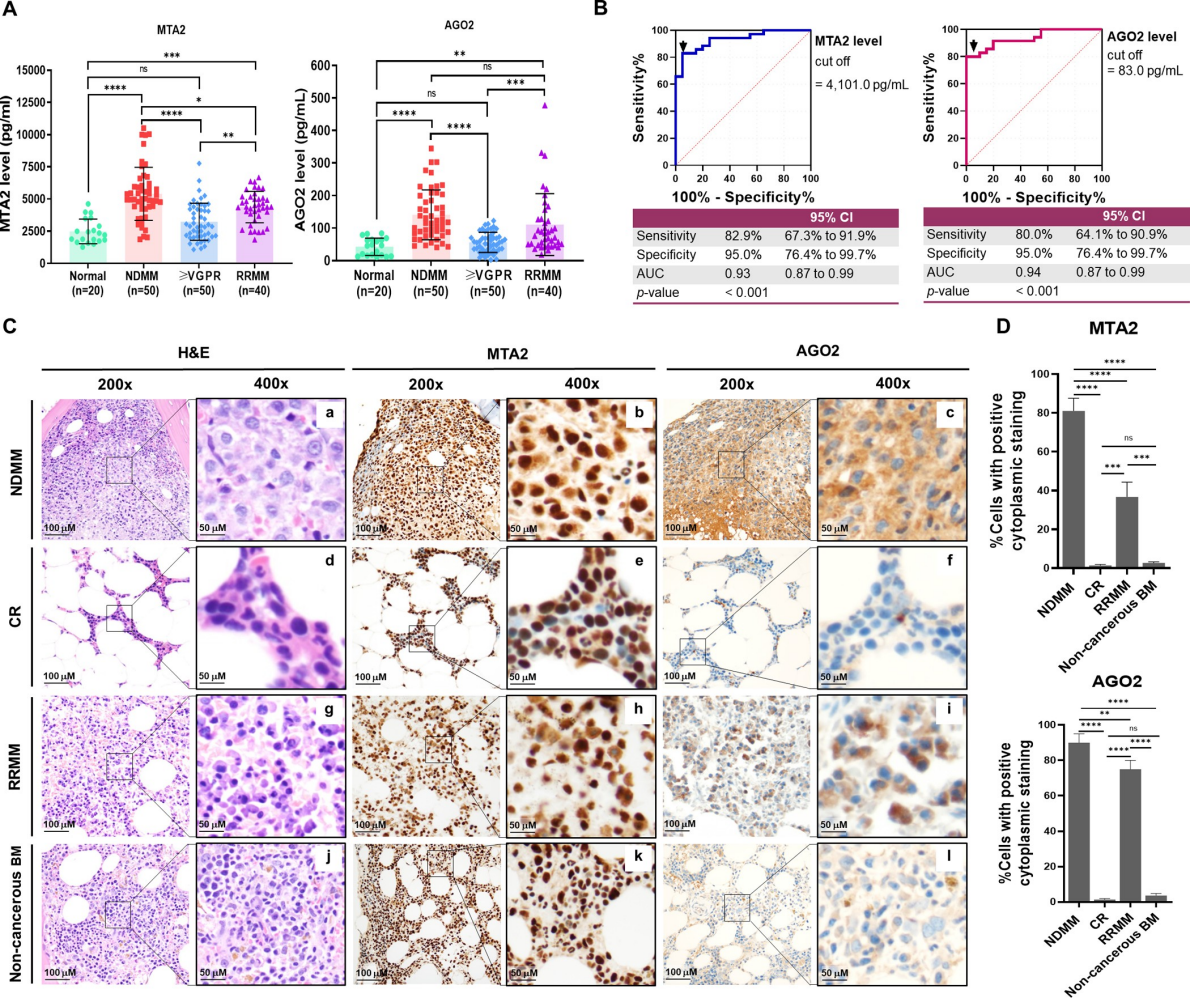
Measuring MTA2 and AGO2 levels in the verification phase. (A) Comparison of serum MTA2 and AGO2 levels among normal control, NDMM, ≥VGPR and RRMM groups (B) The ROC analysis demonstrated the diagnostic performance and provided a specific cut-off for serum MTA2 and AGO2. (C) Protein expression levels of MTA2 and AGO2 by IHC staining in representative NDMM, CR, RRMM and non-cancerous BM core biopsies. The MTA2 protein was localized in the nucleus of all leukocytes (panels e and k, 400x), which was considered an internal positive control. In NDMM and RRMM, MTA2 protein was localized in both the nucleus and cytoplasm of myeloma cells (panels b and h, 400x). The AGO2 protein was mainly localized in the cytoplasm of myeloma cells, especially in NDMM and RRMM (panels c and i, 400x). (D) Relative expressions of MTA2 and AGO2 by IHC in NDMM, CR, RRMM and non-cancerous BM core biopsies. * represents *p* < 0.05, ** represents *p* < 0.01, *** represents *p* < 0.001, **** represents *p* < 0.0001 and ns represents not significant.

MTA2 and AGO2 concentrations were measured using an ELISA assay (Fig 4A). For normal subjects, median (IQR) MTA2 and AGO2 levels were 2,274.0 (1,750.0−3,332.0) pg/mL and 46.19 (15.73−64.17) pg/mL, respectively. The median MTA2 levels found among patients with NDMM, ≥VGPR and RRMM were 5,001.0 (4,356.0−6,069.0), 2,889.0 (2,091.0−4,256.0) and 4,395.0 (3,720.0−5,149.0) pg/mL, respectively. The median AGO2 levels among patients with NDMM, ≥VGPR and RRMM were 123.4 (90.9−208.9), 55.6 (34.0−73.9) and 80.1 (48.2−127.7) pg/mL, respectively. As compared with controls, both MTA2 and AGO2 levels were consistently higher in NDMM and RRMM (*p* < 0.05), whereas no significant difference was observed between ≥VGPR and controls (*p* > 0.05, Fig 4A).

#### ROC analysis, specific cut-off determination

The diagnostic performance and specific cut-off for MM discrimination were analyzed based on MTA2 and AGO2 levels from controls and NDMM. Sensitivity, specificity and ROC with AUC were evaluated. The MTA2 level of 4,101.0 pg/mL could provide a sensitivity of 82.6% and a specificity of 95.0%; while the AGO2 level of 83.0 pg/mL could provide a sensitivity of 80.0% and a specificity of 95.0% (Fig 4B). These levels were considered the optimal specific cut-offs.

#### Protein expression levels of MTA2 and AGO2 by IHC staining

Protein expression levels of MTA2 and AGO2 by IHC staining in representative NDMM, CR, RRMM and non-cancerous BM are shown in Fig 4C. The MTA2 protein was localized in the nucleus of all leukocytes (panels e and k, 400x), which was considered an internal positive control. On the other hand, among patients with MM (NDMM and RRMM), the MTA2 protein was localized in both the nucleus and cytoplasm of myeloma cells (panels b and h, 400x). The AGO2 protein was mainly localized in the cytoplasm of myeloma cells, especially in NDMM and RRMM (panels c and i, 400x). The protein expression by IHC was quantified and shown as a percentage of cells with positive cytoplasmic staining. Relative expressions of MTA2 and AGO2 in NDMM, CR, RRMM and non-cancerous BM are demonstrated in Fig 4D.

### Validation phase

#### Validation of serum MTA2 and AGO2 using paired serum samples from NDMM and RRMM cohorts

**Fig 5:**
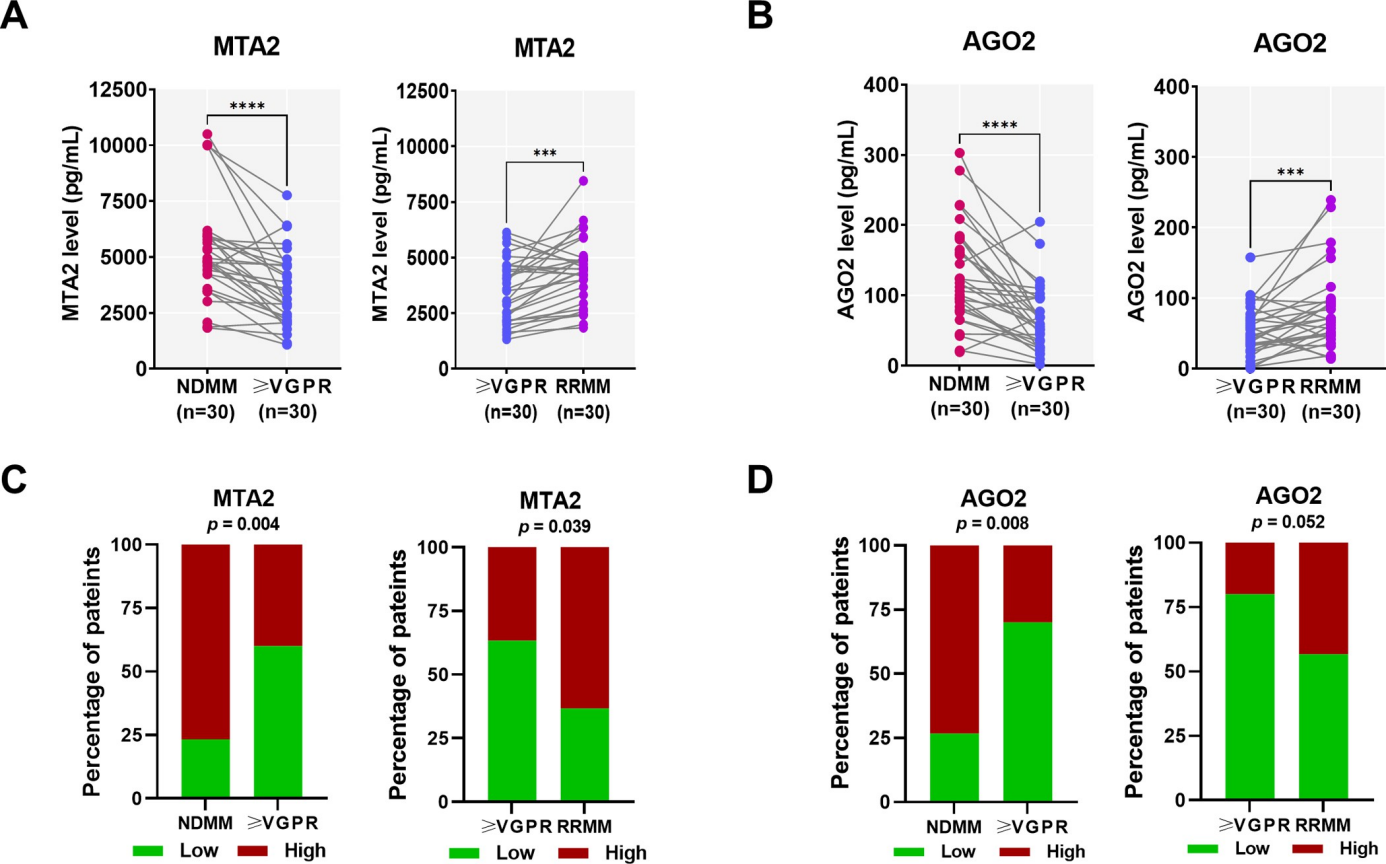
Serum MTA2 and AGO2 levels measured by ELISA in the validation cohorts. Comparison of serum (A) MTA2 and (B) AGO2 levels measured by the ELISA technique in NDMM and RRMM cohorts. * represents *p* < 0.05, ** represents *p* < 0.01, *** represents *p* < 0.001 and **** represents *p* < 0.0001. (C-D) The proportion of patients with high and low MTA2 and AGO2 levels in the NDMM and RRMM cohorts.

In the NDMM cohort, the MTA2 levels measured when newly diagnosed were significantly higher than those measured at ≥VGPR, with a median of 4,825.0 (4,064.0−5,952.0) *vs*. 3,281.0 (2,154.0−4,654.0) pg/mL (*p* < 0.0001, Fig 5A). Consistently, the AGO2 levels measured when newly diagnosed were significantly higher than those measured at ≥VGPR, with a median of 114.9 (78.4−180.4) *vs*. 59.1 (32.8−96.9) pg/mL (*p* < 0.0001, Fig 5B).

In the RRMM cohort, both MTA2 and AGO2 levels measured at ≥VGPR were significantly lower than those measured at the diagnosis of RRMM (*p* < 0.001, Fig 5A and 5B). The median MTA2 levels measured at ≥VGPR and at the diagnosis of RRMM were 3,548.0 (2,181.0−4,620.0) and 4,435.0 (3,226.0−5,012.0) pg/mL, respectively. Also, the median AGO2 levels measured at ≥VGPR and at the diagnosis of RRMM were 48.3 (30.9−73.9) and 70.1 (47.1−96.9) pg/mL, respectively.

#### Association between serum MTA2 and AGO2 levels and disease characteristics

Next, the association between MTA2 and AGO2 levels and disease features of patients with NDMM were assessed. The MTA2 and AGO2 levels at newly diagnosis of MM were used to evaluate the clinical association. Patients were divided in two groups, high and low protein expressions, using the specific cut-offs established for those markers: 4,101.0 and 83.0 pg/mL for MTA2 and AGO2, respectively. Proportions of patients with high and low levels of MTA2 and AGO2 in the NDMM and RRMM cohorts are shown in Fig 5C and 5D. The demographic and disease characteristic data were analyzed and compared using high and low protein expression groups (S4 Table in S1 File).

In terms of age, sex, types of heavy and light chains, the ISS stage, ASCT eligibility, and treatment groups, no difference between patients with high and low MTA2 levels was observed (*p* > 0.05). Low MTA2 levels were associated with hypercalcemia among patients with NDMM (*p* = 0.031). Similarly, no significant difference was found between high and low AGO2 levels in terms of age, ISS stage, ASCT eligibility and treatment group (*p* > 0.05). In contrast, high AGO2 levels were significantly observed among females rather than males (*p* = 0.024) and were associated with IgG isotype (*p* = 0.033), kappa light chains isotype (*p* = 0.039) and bone involvement features (*p* = 0.009, S4 Table in S1 File).

#### Impact of serum MTA2 and AGO2 levels on patient outcomes

Regarding the treatment response and MM disease progression as events of interest, in the NDMM cohort, the TTR was counted from the start of treatment to the first observation of the response of ≥VGPR. The impact of the biomarker levels on patients’ TTR was evaluated using the biomarker levels when newly diagnosed. For the RRMM cohort, the PFS was counted after the patient achieved a response of ≥VGPR until diagnosis for disease progression, and the levels of the biomarkers at ≥VGPR were used to evaluate the impact on patients’ PFS. The OS was not assessed due to the low number of events (deaths) at the time of analysis.

**Fig 6:**
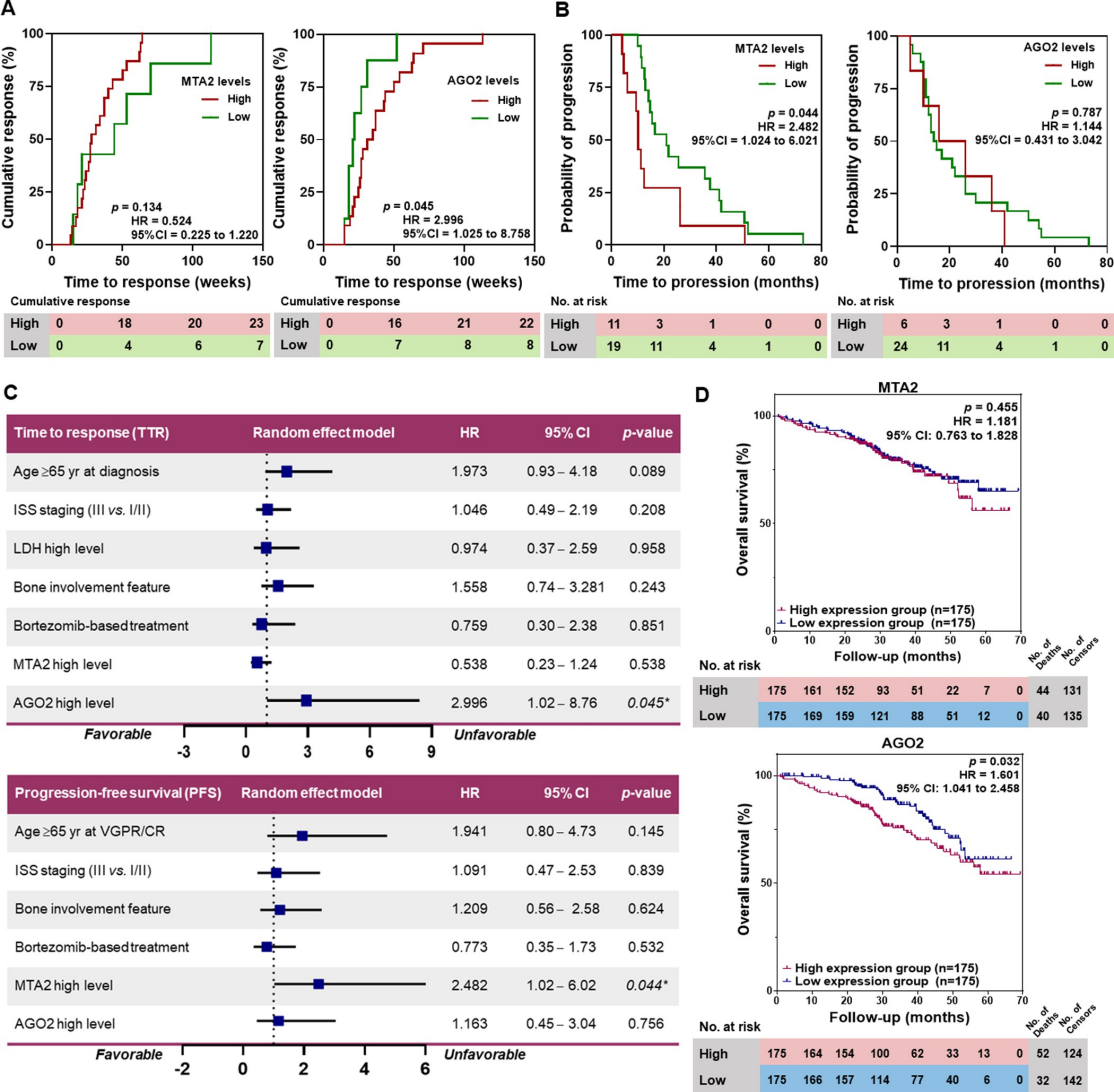
Impact of MTA2 and AGO2 levels on patient outcomes. (A) Kaplan-Meier plots demonstrated the TTR of patients according to MTA2 and AGO2 levels. (B) Kaplan-Meier plots demonstrated the PFS of patients according to MTA2 and AGO2 levels. (C) Forest plots of univariate Cox regression models with probabilities for each factor associated with TTR and PFS of MM based on clinical variables. (D) Kaplan-Meier analysis of patient outcome data from the GSE2658 [28] data set (n = 350) demonstrated the OS of patients with MM expressing higher levels of MTA2 and AGO2 compared with those with lower expression levels based on the median expression of the cohort.

No significant difference in TTR was observed among patients with NDMM with high and low MTA2 levels. Interestingly, when newly diagnosed, high AGO2 levels were associated with prolonged TTR (median TTR 33.0 *vs*. 21.5 weeks, *p* = 0.045, HR = 3.00, 95% CI, 1.02 to 8.76) compared with those with low levels (S5 Table in S1 File and Fig 6A). In contrast, high MTA2 levels at ≥VGPR were associated with shorter PFS (median PFS 10.2 *vs*. 20.9 months, *p* = 0.044, HR = 2.48, 95% CI, 1.02 to 6.02) compared with those with low levels. This effect was not observed at high and low AGO2 levels (S5 Table in S1 File and Fig 6B). Moreover, univariate Cox regression analysis was used to analyze the independent clinical variables associated with TTR and PFS of patients with MM, including age, ISS stage III *vs*. I/II, high LDH level, bone involvement features and treatment regimens. No association was observed between these variables and patients’ TTR and PFS in this study population. This implied that high AGO2 and MTA2 levels were independent factors associated with prolonged TTR and shorter PFS among patients with MM (Fig 6C).

#### Association of *MTA2* and *AGO2* gene expressions and patients with MM survival using the NCBI GEO data set

Furthermore, the effects of *MTA2* and *AGO2* gene expressions on patients with MM survival were evaluated by analyzing GSE2658 [28]. No significant difference in OS was observed between patients with high and low *MTA2* expression levels (Log-rank test *p* = 0.455, HR = 1.18, 95% CI, 0.76 to 1.82). Notably, a significantly reduced OS was observed among patients with high *AGO2* expression levels compared with those with low expression levels (Log-rank test *p* = 0.032, HR = 1.60, 95% CI, 1.04 to 2.46, Fig 6D).

## Discussion

In the present study, LC–MS/MS analysis was used to characterize the serum proteomic profile in each state of MM and to identify potential biomarkers corresponding to MM disease activity. Our results revealed aberrant expressions of 25 upregulated and 13 downregulated proteins among NDMM and RRMM compared to ≥VGPR groups. Subsequently, KEGG enrichment pathway analysis predicted that most dysregulated proteins were associated with several pathways in cancer, including RNA transport, miRNAs and the p53 signaling pathways. The analysis using the NCBI GEO microarray dataset revealed that *MTA2*, *AGO2*, *PRODH* and *TMC1* genes showed significant upregulation, whereas *FAM120C* demonstrated significant downregulation among patients with MM. These results agree with our results from LC–MS/MS. Moreover, MTA2 and AGO2 were selected for further verification and validation studies as candidate biomarkers.

In the verification phase, the serum concentrations of MTA2 and AGO2 measured by ELISA were consistent with the results from LC–MS/MS. Those protein levels were significantly higher in the disease-active states, NDMM and RRMM, compared with ≥VGPR and control. When the diagnostic performance of those biomarkers was evaluated, we found an excellent diagnostic value of both MTA2 and AGO2, with high sensitivity and specificity, to discriminate patients with MM from normal individuals. In addition, IHC staining was performed to confirm the expression of these proteins in myeloma cells. Accumulations of MTA2 and AGO2 in the cytoplasm of myeloma cells were observed in the BM samples collected at NDMM and RRMM. This finding supports our hypothesis that myeloma cells are major sources of these proteins, and the increased levels in blood circulation could indicate disease activity among patients with MM.

In the validation phase, we analyzed the serum levels of these biomarkers using paired serum samples obtained from patients with NDMM and RRMM. Our results confirmed a significant alteration in the serum levels of MTA2 and AGO2 in both NDMM and RRMM cohorts. We further investigated the association between serum MTA2 and AGO2 levels and patient disease characteristics and outcomes. Regardless of the patient treatment regimens, high MTA2 levels measured at ≥VGPR were associated with shorter PFS. High AGO2 levels were frequently observed among females and were associated with IgG and kappa light chain isotypes. Notably, high AGO2 levels were also associated with the occurrence of bone involvement features and were an independent factor associated with prolonged TTR among patients with NDMM. On the contrary, a recent study demonstrated that an elevated level of AGO2 expression was significantly associated with a shorter PFS among patients with MM [29]. However, this finding was not observed in our study.

MTA2 belongs to a member of the metastasis tumor-associated protein family of transcriptional regulators, controlling the organization of the cytoskeleton at the transcriptional level. MTA2 is also a central component of the NuRD complex, playing a transcriptional regulatory role via histone deacetylation and chromatin remodeling [30]. MTA2 overexpression has been reported in various human cancers, including gastrointestinal, lung, renal, breast and hepatocellular carcinoma. Moreover, its overexpression is associated with tumor invasion capacity, metastasis and an unfavorable prognosis [30–35]. In hematological malignancies, studies relating to MTA2 remain limited. A large sample size study using the whole-genome sequencing (WGS) technique revealed an association of mutation on the MTA2 gene with abnormal clonal hematopoiesis but this mutation has no known involvement in myeloid neoplasia [36]. A recent animal model study demonstrated that the loss of MTA2 leads to BM and splenic B–cell developmental defects in mice [37]. However, studying the MTA2 role and function involved in MM is required.

The other protein, AGO2 or EIF2C2, a member of the argonaute protein family, plays an important role in regulating epigenetic gene expression, miRNAs function and maturation [38, 39]. AGO2 of the human AGO protein family is the only member with intrinsic endoribonuclease activity, which is essential for a nonredundant slicer-independent function within the mammalian miRNA pathway [40]. AGO2 overexpression has been reported in several carcinomas, including breast, head and neck squamous cell, nasopharyngeal, urothelial, ovarian and colorectal carcinomas [41–46]. In MM, dysregulated AGO2 expression contributes to MM pathogenesis, myeloma cell growth and survival, apoptosis, angiogenesis and drug resistance mechanisms [47–52]. A related study reported an association between the global elevation of miRNAs and the overexpression of AGO2 in high-risk patients with MM [53]. Moreover, the analysis results in our study, using data from the GSE2658 [28] dataset, revealed a significant association between AGO2 overexpression and shorter OS among patients with MM.

In clinical practice, the diagnosis and monitoring of MM disease status are highly dependent on BM examination, involving an invasive and painful procedure. Hence, implementing serum MTA2 and AGO2 detection may be beneficial due to its simplicity, shortening turnaround time, being less painful and being performable in a simple laboratory. Moreover, the serum levels of these proteins demonstrated prognostic value and could serve as a better indicator for monitoring and predicting adverse outcomes among patients with MM.

Our study encountered some limitations. First, this constituted a single-center study with a relatively small number of patients in the validation cohorts. Second, cytogenetic abnormality plays a pivotal role in MM pathogenesis, which may have affected protein expression profiles. Owing to data inadequacy, the associations of MTA2 and AGO2 levels with cytogenetic abnormality were not evaluated in this study. Therefore, further studies with larger sample sizes, completed cytogenetic profiles and analysis of these two biomarkers compared with traditional biomarkers such as SFLC for disease monitoring are suggested.

In conclusion, this study demonstrated the proteomic approach to characterizing and identifying serum biomarkers among patients with MM. Interestingly, MTA2 and AGO2 proteins were first identified as potential serum biomarkers providing prognostic value and potential in clinical applications. Elevated levels of these biomarkers correlated with disease activity and were associated with adverse outcomes among patients with MM. Nevertheless, validation and standardization of the proposed biomarkers before implementing in clinical practice are recommended.

## Supporting information

S1 File (DOCX)
