# Supplementary material for: Serum proteomic profiling reveals MTA2 and AGO2 as potential prognostic biomarkers associated with disease activity and adverse outcomes in multiple myeloma
Source: PLoS One. 2022 Dec 1;17(12):e0278464. doi: 10.1371/journal.pone.0278464 (PMC9714744; doi:10.1371/journal.pone.0278464)
Supplement: S1 File — (DOCX) [file pone.0278464.s001.docx]

**SUPPLEMENTARY MATERIAL**

**Serum proteomic profiling reveals MTA2 and AGO2 as potential prognostic biomarkers associated with disease activity and adverse outcomes in multiple myeloma**

Dollapak Apipongrat^1, 2^, Sittiruk Roytrakul^3^, Kannadit Prayongratana^2^, Mongkon Charoenpitakchai^4^, Kamphon Intharanut^1^, Chonlada Laoruangroj^2^, Panachai Silpsamrit^2^, Oytip Nathalang^1*^

^1^Graduate Program in Biomedical Sciences, Faculty of Allied Health Sciences, Thammasat University, Pathumthani, ^2^Division of Hematology, Department of Medicine, Phramongkutklao Hospital, Bangkok, ^3^Functional Ingredients and Food Innovation Research Group, National Center for Genetic Engineering and Biotechnology, National Science and Technology Development Agency, Pathumthani, ^4^Department of Pathology, Pramongkutklao College of Medicine, Bangkok, Thailand

**Running title**: MTA2 and AGO2 as potential prognostic biomarkers in MM

**Correspondence:** Prof. Oytip Nathalang, Ph.D.

Graduate Program in Biomedical Sciences, Faculty of Allied Health Sciences, Thammasat University, Pathumthani 12120 Thailand.

Tel: +662-9869213, Fax: +662-5165379

E-mail: oytipntl@hotmail.com

**SUPPLEMENTARY MATERIAL**

**List of contents**

**Supplementary Methods**

1. Proteomic analysis using Nano-LC-MS/MS

2. Enzyme-linked immunosorbent assay (ELISA)

3. Immunohistochemical (IHC) staining

**Supplementary Tables**

**S1 Table** Demographics and disease characteristics of 375 patients and 70 age-matched normal controls included in the discovery phase

**S2 Table** Patient demographics and baseline disease characteristics of 60 patients in validation cohorts

**S3a Table** The significant upregulated proteins identified in NDMM and RRMM serum samples with a log_2_-fold change ([NDMM and RRMM] – [≥VGPR]) > 1.5.

**S3b Table** The significant downregulated proteins identified in NDMM and RRMM serum samples with log_2_-fold change ([NDMM and RRMM] – [≥VGPR]) < -1.5.

**S4 Table** Association between patient’s demographics and disease characteristics and the proteins levels in patients with NDMM

**S5 Table** Univariate Cox-regression analysis of the TTR and PFS in patients with MM based on MTA2 and AGO2 levels

**Supplementary Methods**

**1. Proteomic analysis using Nano-LC-MS/MS**

**1.1 Sample preparation and protein digestion**

The protein concentration of all serum samples was determined by Lowry assay using bovine serum albumin (BSA) as a standard protein [1]. Five micrograms of protein samples were subjected to in-solution digestion. Samples are completely dissolved in 10 mM ammonium bicarbonate (AMBIC). To reduce disulfide bonds, 5 mM dithiothreitol (DTT) in 10 mM AMBIC is added, then incubated at 60ºC for 1 hour, followed by alkylation of sulfhydryl groups by using 15 mM iodoacetamide (IAA) in 10 mM AMBIC at room temperature for 45 minutes in the dark. For digestion, samples are mixed with 50 ng/µl of sequencing grade Trypsin (Promega, Germany) by a 1:20 ratio and incubated at 37ºC overnight. The digested samples must be dried and resuspended with 0.1 % formic acid before injection into LC-MS/MS.

**1.2 Liquid Chromatography-Tandem Mass Spectrometry (LC/MS-MS)**

The tryptic peptide samples were prepared for injection into an Ultimate3000 Nano/Capillary LC System (Thermo Scientific, UK) coupled to a Hybrid Quadrupole Q-Tof Impact II™ (Bruker Daltonics, Germany) equipped with a Nano-captive spray ion source. Briefly, 1 μL of peptide digests were enriched on a µ-Precolumn 300 µm i.d. × 5 mm C18 Pepmap100, 5 µm, 100 ºA (Thermo Scientific, UK), separated on a 75 μm I.D. × 15 cm, and packed with Acclaim PepMap RSLC C18, 2 μm, 100 ºA, nanoViper (Thermo Scientific, UK). The C18 column was enclosed in a thermostatted column oven set to 60°C. Solvent A and B containing 0.1% formic acid in water and 0.1 % formic acid in 80% acetonitrile, respectively are supplied on the analytical column. A gradient of 5–55% solvent B was used to elute the peptides at a constant flow rate of 0.30 μL/min for 30 minutes. Electrospray ionization is carried out at 1.6 kV using the CaptiveSpray. Nitrogen was used as a drying gas (flow rate about 50 L/h). Collision-induced-dissociation (CID) product ion mass spectra were obtained using nitrogen gas as the collision gas. Mass spectra and MS/MS spectra were obtained in the positive-ion mode at 2 Hz over the range (*m/z*) 150–2200. The collision energy was adjusted to 10 eV as a function of the *m/z* value. The LC-MS analysis of each sample was done in triplicate.

**1.3 Bioinformatics and label-free quantitation**

MaxQuant 1.6.6.0 was used to quantify the proteins in individual samples using the Andromeda search engine to correlate MS/MS spectra to the Uniprot *Homo sapiens* database [2]. Label-free quantitation with MaxQuant's standard settings was performed: maximum of two miss cleavages, a mass tolerance of 0.6 Da for the main search, trypsin as digesting enzyme, carbamidomethylation of cysteine as fixed modification, and the oxidation of methionine and acetylation of the protein N-terminus as variable modifications. Only peptides with a minimum of 7 amino acids, as well as at least one unique peptide, are required for protein identification. Only proteins with at least two peptides, and at least one unique peptide, were considered as being identified and used for further data analysis. The protein false discovery rate (FDR) was set at 1% and estimated by using the reversed search sequences. The maximal number of modifications per peptide is set to 5. As a search FASTA file, the proteins presented in the *Homo sapiens* proteome were downloaded from Uniprot. Potential contaminants presented in the contaminants.fasta file that comes with MaxQuant were automatically added to the search space by the software.

The MaxQuant ProteinGroups.txt file was loaded into Perseus version 1.6.6.0 [3], potential contaminants that did not correspond to any UPS1 protein are removed from the data set. Maximum peptide intensities are log_2_ transformed and missing values are also imputed in Perseus using constant value (zero). The visualization and statistical analyses are conducted using the MultiExperiment Viewer (MeV) in the TM4 suite software [4] .

To explore the potential functions of differentially expressed proteins and the pathways these proteins might be involved, the Protein Analysis Through Evolutionary Relationships (PANTHER version 11.1, available from: http://www.pantherdb.org/) analysis was performed by keeping the *Homo sapiens* as a selected organism [5]. Bioinformatics analyses, including Gene Ontology (GO) annotation and Kyoto Encyclopedia of Genes and Genomes (KEGG) pathway and protein domain functional enrichment, were performed to classify the functions of the identified proteins. When FDR was <1.0%, GO terms and KEGG pathways were significantly enriched. In addition, a protein-protein interaction (PPI) network was constructed using STITCH version 5.0 (available from: http://stitch.embl.de/) to analyze the common and the forecasted functional interaction networks between identified proteins and small molecules [6].

**2. Enzyme-linked immunosorbent assay (ELISA)**

The concentrations of MTA2 and AGO2 in serum samples were measured using commercial ELISA kits (Human MTA2 [MBS2705865] and Human Proteins Argonnaute-2/EIF2C2 [MBS910054] ELISA kits, MyBioSource, CA, USA). The assay procedure was performed according to the instructions of manufacturer.

**2.1 Human MTA2 ELISA kit**

The test principle applied in this kit is sandwich enzyme immunoassay. The microtiter plate provided in this kit has been pre-coated with an antibody specific to MTA2. Standards or samples are then added to the appropriate microtiter plate wells with a biotin-conjugated antibody specific to MTA2. Next, avidin conjugated to horseradish peroxidase (HRP) is added to each microplate well and incubated. After 3,3',5,5'-Tetramethylbenzidine (TMB) substrate solution is added, only those wells that contain MTA2, biotin-conjugated antibody and enzyme-conjugated avidin will exhibit a change in color. The enzyme-substrate reaction is terminated by the addition of sulphuric acid solution and the color change is measured spectrophotometrically at a wavelength of 450 nm ± 10 nm. The concentration of MTA2 in the samples is then determined by comparing the optical density (O.D.) of the samples to the standard curve.

The assay procedure is following;

1.) Determine wells for diluted standard, blank and sample. Prepare 7 wells for standard, 1 well for blank. Add 100 μL each of dilutions of standard, blank and samples into the appropriate wells. Cover with the plate sealer. Incubate for 1 hour at 37ºC.

2.) Remove the liquid of each well, don’t wash.

3.) Add 100 μL of Detection Reagent A working solution to each well, cover the wells with the plate sealer and incubate for 1 hour at 37ºC.

4.) Aspirate the solution and wash with 350 μL of 1× Wash Solution to each well using a squirt bottle, multi-channel pipette, manifold dispenser or autowasher, and let it sit for 1~2 minutes. Remove the remaining liquid from all wells completely by snapping the plate onto absorbent paper. Totally wash 3 times. After the last wash, remove any remaining Wash Buffer by aspirating or decanting. Invert the plate and blot it against absorbent paper.

5.) Add 100 μL of Detection Reagent B working solution to each well, cover the wells with the plate sealer and incubate for 30 minutes at 37ºC.

6.) Repeat the aspiration/wash process for total 5 times as conducted in step 4.

7.) Add 90 μL of Substrate Solution to each well. Cover with a new plate sealer. Incubate for 10-20 minutes at 37ºC (Don't exceed 30 minutes). Protect from light. The liquid will turn blue by the addition of Substrate Solution.

8.) Add 50 μL of Stop Solution to each well. The liquid will turn yellow by the addition of Stop solution. Mix the liquid by tapping the side of the plate. If color change does not appear uniform, gently tap the plate to ensure thorough mixing.

9.) Remove any drop of water and fingerprint on the bottom of the plate and confirm there is no bubble on the surface of the liquid. Then, run the microplate reader and conduct measurement at 450 nm immediately.

**2.2 Human Proteins Argonnaute-2/EIF2C2**

This assay employs the quantitative sandwich enzyme immunoassay technique. Antibody specific for AGO2 or EIF2C2 has been pre-coated onto a microplate. Standards and samples are pipetted into the wells and any AGO2 present is bound by the immobilized antibody. After removing any unbound substances, a biotin-conjugated antibody specific for AGO2 is added to the wells. After washing, avidin conjugated HRP is added to the wells. Following a wash to remove any unbound avidin-enzyme reagent, a substrate solution is added to the wells and color develops in proportion to the amount of AGO2 bound in the initial step. The color development is stopped and the intensity of the color is measured.

The assay procedure is following;

1.) Add 100 μL of standard and sample per well. Cover with the adhesive strip. Incubate for 2 hours at 37°C.

2.) Remove the liquid of each well, don’t wash.

3.) Add 100 μL of Biotin-antibody (1x) to each well. Cover with a new adhesive strip. Incubate for 1 hour at 37°C. Biotin-antibody (1x) may appear cloudy. Warm up to room temperature and mix gently until solution appears uniform.

4.) Aspirate each well and wash, repeating the process two times for a total of three washes. Wash by filling each well with 200 μL of Wash Buffer using a squirt bottle, multi-channel pipette, manifold dispenser, or autowasher, and let it stand for 2 minutes, complete removal of liquid at each step is essential to good performance. After the last wash, remove any remaining wash buffer by aspirating or decanting. Invert the plate and blot it against clean paper towels.

5.) Add 100 μl of HRP-avidin (1x) to each well. Cover the microtiter plate with a new adhesive strip. Incubate for 1 hour at 37°C.

6.) Repeat the aspiration/wash process for five times as in step 4.

7.) Add 90 μL of TMB Substrate to each well. Incubate for 15-30 minutes at 37°C. Protect from light.

8.) Add 50 μL of Stop Solution to each well, gently tap the plate to ensure thorough mixing.

9.) Determine the O.D. of each well within 5 minutes, using a microplate reader set to 450 nm. If wavelength correction is available, set to 540 nm or 570 nm. Subtract readings at 540 nm or 570 nm from the readings at 450 nm. This subtraction will correct for optical imperfections in the plate. Readings made directly at 450 nm without correction may be higher and less accurate

**3. Immunohistochemical (IHC) staining**

The IHC staining was performed on a Ventana Discovery XT automated system (Ventana Medical System, Tucson, AZ, USA) using the primary antibody raised against MTA2 (diluted 1:500; ab8106, Abcam, UK) AGO2 (diluted 1:200; Ab226943, Abcam, UK). For all subsequent steps, the manufacturer’s reagents, buffers, and instructions were followed for staining. The recommended protocol for immunohistochemistry staining using Ventana Discovery XT is described below

**3.1 Deparaffinization**

Paraffin sections of 4 µm thickness are baked overnight at 50°C. The deparaffinization step is done in the Ventana Discovery XT platform using EZ prep solution.

**3.2 Standard antigen retrieval method**

The standard antigen retrieval method is Heat Induced Epitope Retrieval (HIER) in Tris-EDTA buffer pH 7.8 at 95°C for 44 min (standard Cell Conditioning 1, CC1) and is performed in the Ventana Discovery XT. If expected results are not achieved, test a different time or use Citrate-based buffer pH 6.0 or Protease antigen retrieval instead.

**3.3 Blocking steps**

For blocking endogenous peroxides and protein, these steps are done in the Ventana Discovery XT platform using Inhibitor CM.

**3.4 IHC staining protocol Ventana Discovery XT**

The assay procedure is following;

1.) Deparaffinization in EZ prep 75°C 8 minutes.

2.) Cell Conditioning using Conditioner #1, Standard CC1, 95°C 44 minutes.

3.) Block with Inhibitor CM, 37°C 4 minutes. No Heat.

4.) Incubation with 200 uL of primary antibody for 60 minutes.

5.) Apply one drop of OmniMap anti-Rb HRP and incubate for 16 minutes.

6.) Apply one drop of 3, 3'-diaminobenzidine (DAB CM) and one drop of H_2_O_2_, incubate 8 minutes.

7.) Apply one drop of Copper CM, incubate 5 minutes.

8.) Counterstain with Hematoxylin, incubate for 8 minutes.

9.) Post counterstain with Bluing Reagent, incubate for 8 minutes.

Wash the slides in warm tap water with detergent and dehydrate in graded ethanol and xylene. Coverslip the slides in permanent mounting media.

**Supplementary Tables**

**S1 Table** Demographics and disease characteristics of 375 patients and 70 age-matched normal controls included in the discovery phase

| **Characteristics** | **Study group** | | | |
| --- | --- | --- | --- | --- |
|  | **Normal control**  **(n=70)** | **NDMM**  **(n=57)** | **≥VGPR**  **(n=228)** | **RRMM**  **(n=90)** |
| Age (years), mean±SD | 65.4±10.2 | 65.8±10.4 | 57.0±10.2 | 64.6±10.8 |
| Sex, n (%)  Male  Female | 37 (52.9)  33 (47.1) | 37 (64.9)  20 (35.1) | 124 (54.4)  104 (45.6) | 67 (74.4)  23 (25.6) |
| Heavy chain type, n (%)  IgG  IgA  Other (IgM, IgD, and IgE)  None (light chain myeloma) | N/A  N/A  N/A  N/A | 34 (59.6)  18 (31.6)  1 (1.8)  4 (7.0) | 135 (59.2)  53 (23.2)  2 (0.9)  38 (16.7) | 68 (75.6)  18 (20.0)  0 (0.0)  4 (4.4) |
| Light chain type, n (%)  Kappa (κ)  Lambda (λ) | N/A  N/A | 36 (63.2)  21 (36.8) | 118 (51.8)  110 (48.2) | 43 (47.8)  47 (52.2) |
| ISS, n (%)  I  II  III | N/A  N/A  N/A | 10 (17.5)  14 (24.6)  33 (57.9) | 30 (13.2)  20 (8.8)  178 (78.0) | 8 (8.9)  16 (17.8)  66 (73.3) |

Ig, immunoglobulin; IQR, interquartile range; ISS, International Staging System; NDMM, newly diagnosed multiple myeloma; RRMM, relapsed/refractory multiple myeloma; SD, standard deviation; ≥VGPR, patients with MM who achieved the response at least very good partial response to treatment

**S2** **Table** Patient demographics and baseline disease characteristics of 60 patients in validation cohorts

| **Characteristic** | **NDMM cohort**  **(*n* = 30)** | **RRMM cohort**  **(*n* = 30)** |
| --- | --- | --- |
| Age, year  Mean±SD | 58.93±11.96 | 62.20±10.01 |
| Sex, *n* (%)  Male  Female | 16 (53.3)  14 (46.7) | 20 (66.7)  10 (33.3) |
| Heavy chain type, *n* (%)  IgG  IgA  Other (IgM, IgD, and IgE)  None (light chain myeloma) | 13 (43.3)  11 (36.7)  0 (0.0)  6 (20.0) | 24 (80.0)  6 (20.0)  0 (0.0)  0 (0.0) |
| Light chain type, *n* (%)  Kappa (κ)  Lambda (λ) | 17 (56.7)  13 (43.3) | 18 (60.0)  12 (40.0) |
| ISS stage, *n* (%)  I or II  III | 14 (46.7)  16 (53.3) | 7 (23.3)  23 (76.7) |
| ASCT, *n* (%)  ASCT eligible (≤65 yrs.)  Non-ASCT eligible (>65 yrs.) | 20 (66.7)  10 (33.3) | 20 (66.7)  10 (33.3) |
| First-line treatment regimen, *n* (%)  Bortezomib based  Non-Bortezomib based | 25 (83.3)  5 (16.7) | 23 (76.7)  7 (23.3) |
| Following time, days  Median (range), | 194 (97−793) | 456 (119−2,192) |

ASCT, autologous stem cell transplantation; Ig, immunoglobulin; IQR, interquartile range; ISS, International Staging System; NDMM, newly diagnosed multiple myeloma; RRMM, relapsed/refractory multiple myeloma, SD, standard deviation

**S3a Table** The significant upregulated proteins identified in NDMM and RRMM serum samples with a log_2_-fold change ([NDMM and RRMM] – [≥VGPR]) > 1.5.

| **No.** | **Accession No.** | **Gene name** | **Peptide sequence** | **Log_2_-FC** | **p-value** |
| --- | --- | --- | --- | --- | --- |
| 1 | Q8TDI8 | *TMC1* | ESLRPKRK | 7.99 | 0.001 |
| 2 | A0A024QZH0 | *PLA2G4C* | QEWDLAKSLQKTIQAAR | 7.61 | 0.024 |
| 3 | A0A0G2JRT2 | *MUC20* | ASPTIVPHPGDSSASSESR | 6.96 | <0.001 |
| 4 | A0A024R534 | ***MTA2*** | AECSIRLPKAAK | **6.78** | **<0.001** |
| 5 | E7EVV3 | *SPATA18* | QLQNIEEEAALLSIAR | 6.19 | 0.005 |
| 6 | F8W9W0 | *EPHA5* | IPIRWTAPEAIAFR | 6.04 | 0.002 |
| 7 | X5D7N2 | *PRODH* | HALLAPWACRLLVLLR | 5.50 | 0.003 |
| 8 | Q6ZNE1 | *EIF3A* | DRPDLSAPESLQLYLDTR | 5.43 | <0.001 |
| 9 | B3KY41 | *SMNT* | ENWLHSQQREAEQR | 5.33 | 0.017 |
| 10 | Q9UKV8 | ***AGO2*** | AALAQHRDGNSQGSTHSGE-ASDHK | **5.02** | **0.024** |
| 11 | P51587 | *BRCA2* | CLSPERVLLQLKYR | 4.48 | 0.007 |
| 12 | Q86XN6 | *ZNF761* | CEECDKAFHFK | 3.85 | 0.006 |
| 13 | A0A2R8YG28 | *OTOA* | ISPIEIGLFISYDNATK | 3.75 | 0.007 |
| 14 | A0A090N8Y2 | *ERP70* | DIASDQTSGQVDMMHR | 3.41 | 0.019 |
| 15 | O14917 | *PCDH17*  *PCDH68*  *PCH68* | GGGGLQPHSYEARICNYR | 3.35 | 0.005 |
| 16 | Q5SXM1 | *ZNF678* | AGCEECPYKPEGSHK | 3.17 | 0.014 |
| 17 | F5H450 | *FZD10* | ADVHRAGLYPHPR | 3.07 | 0.015 |
| 18 | Q14CC5 | *TPH2* | ELSKLYPTHACR | 2.92 | 0.043 |
| 19 | Q7KZN9 | *COX15* | APARAPALLPLYRQK | 2.35 | 0.003 |
| 20 | A0A0C4DG03 | *SULT6B1* | GFLFPGVAGHTDQK | 2.25 | 0.024 |
| 21 | Q7Z6I6 | *ARHGAP30* | AYFRELPDPLLTYR | 1.87 | 0.010 |
| 22 | J3KN75 | *TBC1D8B* | EDDPEKFR | 1.73 | 0.002 |
| 23 | A0A024RAC0 | *LUZP1* | AANGLEADNSCPNSK | 1.57 | 0.023 |

**S3b** **Table** The significant downregulated proteins identified in NDMM and RRMM serum samples with a log_2_-fold change ([NDMM and RRMM] – [≥VGPR]) < -1.5.

| **No.** | **Accession No.** | **Gene name** | **Peptide sequence** | **Log2-FC** | **p-value** |
| --- | --- | --- | --- | --- | --- |
| 1 | Q9HCE0 | *EPG5* | DWPKRLYTSHFAYLIFEPK | -1.94 | 0.001 |
| 2 | Q9UKT9 | *IKZF3* | AEMSNGAPQELEKK | -2.16 | 0.008 |
| 3 | Q13470 | *MKRN2* | AQEPDFPHLVQRLCIECVEG-HLYVCADGR | -2.28 | 0.001 |
| 4 | O60500 | *NPHS1* | KSLILNVK | -2.81 | 0.000 |
| 5 | C6KE32 | *P2RX7* | CCQPCVVNEYYYR | -2.94 | 0.001 |
| 6 | Q9ULX3 | *NOB1*  *PSMD8BP1* | TFCGHRCLIYR | -3.07 | 0.001 |
| 7 | Q9NX05 | *FAM120C*  *CXorf17* | DRLAEWGRR | -3.11 | 0.031 |
| 8 | A0A0S2Z4T5 | *RIN1* | LELEQVRQKLLQLLR | -3.26 | 0.033 |
| 9 | Q9BV57 | *ADI1*  *MTCBP1 HMFT1638* | DKLPNYEEKIK | -3.66 | 0.006 |
| 10 | A0A1W2PRI9 | *C4orf50* | AEWGSHKWQAFSWNER | -4.24 | 0.008 |
| 11 | Q9BYV9 | *BACH2* | AGDVEMDRK | -4.31 | 0.006 |
| 12 | A0A140VJV5 | *CBY2* | KDVVELSASKDHLLPSPR | -4.79 | 0.004 |
| 13 | B4E100 | *APBB1* | ESKETNEKMNAK | -4.89 | 0.014 |
| 14 | P20848 | *SERPINA2* | AEELHPAGTAETK | -6.25 | 0.002 |
| 15 | Q9BZZ2 | *SIGLEC1* | ADTGFYFCEVQNVHGSER | -6.53 | 0.016 |

**S4 Table** Association between patient’s demographics and disease characteristics and the proteins levels in patients with NDMM

| **Characteristic** | **Serum MTA2 levels** | | | **Serum AGO2 levels** | | |
| --- | --- | --- | --- | --- | --- | --- |
|  | Low  (n=7) | High  (n=23) | p-value | Low  (n=8) | High  (n=22) | p-value |
| Age, Mean±SD | 56.0±15.1 | 59.8±11.1 | 0.465 | 61.0±13.5 | 58.2±11.6 | 0.577 |
| Sex, *n* (%)  Male  Female | 6 (85.7)  1 (14.3) | 10 (43.5)  13 (56.5) | 0.051 | 7 (87.5)  1 (12.5) | 9 (40.9)  13 (59.1) | 0.024* |
| Heavy chain type, *n* (%)  IgG  IgA  None | 4 (57.1)  3 (42.9)  0 (0.0) | 9 (39.1)  8 (34.8)  6 (26.1) | 0.851 | 1 (12.5)  5 (62.5)  2 (25.0) | 12 (54.5)  6 (27.3)  4 (18.2) | 0.033* |
| Light chain type, *n* (%)  Kappa (κ)  Lambda (λ) | 5 (71.4)  2 (28.6) | 12 (52.2)  11 (48.8) | 0.368 | 7 (87.5)  1 (12.5) | 10 (45.5)  12 (54.5) | 0.039* |
| ISS stage, *n* (%)  I or II  III | 3 (42.8)  4 (57.2) | 11 (47.8)  12 (52.2) | 0.818 | 5 (62.5)  3 (37.5) | 9 (40.9)  13 (59.1) | 0.295 |
| ASCT, *n* (%)  ASCT eligible  Non-ASCT eligible | 5 (71.5)  2 (28.5) | 15 (65.2)  8 (34.8) | 0.760 | 4 (50.0)  4 (50.0) | 16 (72.7)  6 (27.2) | 0.243 |
| Hypercalcemia, *n* (%)  Yes  No | 3 (42.9)  4 (57.1) | 1 (4.4)  22 (95.6) | 0.031* | 2 (25.0)  6 (75.0) | 2 (9.1)  20 (90.9) | 0.284 |
| Renal failure, *n* (%)  Yes  No | 1 (14.3)  6 (85.7) | 4 (17.4)  19 (82.6) | 1.000 | 0 (0.0)  8 (100.0) | 5 (22.7)  17 (77.3) | 0.304 |
| Anemia, *n* (%)  Yes  No | 4 (57.1)  3 (42.8) | 17 (73.9)  6 (26.1) | 0.640 | 4 (50.0)  4 (50.0) | 17 (77.3)  5 (22.7) | 0.195 |
| Bone involvements, *n* (%)  Yes  No | 3 (42.8)  4 (57.2) | 16 (69.6)  7 (30.4) | 0.199 | 2 (25.0)  6 (75.0) | 17 (77.3)  5 (22.7) | 0.009* |

*, significance p-value; ASCT, autologous stem cell transplantation; Ig, immunoglobulin; ISS, International Staging System; NDMM, newly diagnosed multiple myeloma; RRMM, relapsed/refractory multiple myeloma, SD, standard deviation

**S5 Table** Univariate Cox-regression analysis of the TTR and PFS in patients with MM based on MTA2 and AGO2 levels

| **Biomarker** | **Group** | **n** | **Time to response (TTR)** | | |  | **Progression-free survival (PFS)** | | |
| --- | --- | --- | --- | --- | --- | --- | --- | --- | --- |
|  |  |  | HR (95% CI) | Median TTR (range), weeks | p-value | **n** | HR (95% CI) | Median PFS (range), months | p-value |
| MTA2 | High | 23 | 0.52 (0.23−1.22) | 28.0 (13.0−64.0) | 0.145 | 11 | 2.48 (1.02−6.02) | 10.2 (4.0−50.8) | 0.044* |
|  | Low | 7 |  | 44.0 (15.0−113.0) |  | 19 |  | 20.9 (9.9−73.1) |  |
| AGO2 | High | 22 | 3.00 (1.03−8.76) | 33.0 (15.0−113.0) | 0.045* | 6 | 1.14 (0.43−3.04) | 21.0 (11.5−26.0) | 0.787 |
|  | Low | 8 |  | 21.5 (15.0−52.0) |  | 24 |  | 14.5 (11.3−29.0) |  |

*, significance p-value; HR, hazard ratio; CI, confidence interval

**References**

1. Lowry O, Rosebrough N, Farr AL, Randall R. Protein measurement with the folin phenol reagent. J Biol Chem. 1951;193(1):265-75.

2. Tyanova S, Temu T, Cox J. The MaxQuant computational platform for mass spectrometry-based shotgun proteomics. Nat Protoc. 2016;11(12):2301-19.

3. Tyanova S, Temu T, Sinitcyn P, Carlson A, Hein MY, Geiger T, et al. The Perseus computational platform for comprehensive analysis of (prote)omics data. Nat Methods. 2016;13:731-40.

4. Howe EA, Sinha R, Schlauch D, Quackenbush J. RNA-Seq analysis in MeV. Bioinformatics. 2011;27(22):3209-10.

5. Mi H, Muruganujan A, Ebert D, Huang X, Thomas PD. PANTHER version 14: more genomes, a new PANTHER GO-slim and improvements in enrichment analysis tools. Nucleic Acids Res. 2019;47(D1):D419-D26.

6. Szklarczyk D, Santos A, von Mering C, Jensen LJ, Bork P, Kuhn M. STITCH 5: augmenting protein–chemical interaction networks with tissue and affinity data. Nucleic Acids Res. 2016;44(D1):D380-D4.
